# Supratonsillar Approach to Deep Cerebellar Cavernous Malformations Near the Dentate Nucleus: 3-Dimensional Operative Video

**DOI:** 10.1093/ons/opx030

**Published:** 2017-05-10

**Authors:** Arnau Benet, Jae Seung Bang, Halima Tabani, Ali Tayebi Meybodi, Michael T Lawton

**Affiliations:** Department of Neurosurgery, University of California, San Francisco, California

Cavernous malformations in or around the dentate nucleus are rare and require transgression of overlying cerebellum to access them from the cortical surface. The supratonsillar approach was introduced previously as an alternative route that avoids tissue transgression by splitting the tonsillobiventral fissure and working through a natural subarachnoid corridor.^1^ This video demonstrates this underutilized supratonsillar approach for microsurgical resection of a deep cerebellar cavernous malformation. A 25-year-old man presented with headaches. Neuroimaging revealed a 10-mm diameter cavernous malformation adjacent to the right dentate nucleus. The patient opted for surgical treatment and consented for surgery. The patient was positioned prone, with neck flexion and a midline posterior nuchal incision was placed. Next, a suboccipital craniotomy and supratonsillar approach were performed. The lesion was exposed through the tonsillobiventral fissure, following the posterior inferior cerebellar artery (PICA) cortical branches and the retro- and supratonsillar veins into the supratonsillar recess. The lesion was removed completely and postoperative imaging confirmed complete resection. The patient recovered well from surgery.

The supratonsillar approach provides direct access to the region of the dentate nucleus and inferior cerebellar peduncle without cerebellar transgression or damage to the vermis. The distal PICA branches, supratonsillar veins, and intersecting folia of the tonsil and biventral lobule are important landmarks for recognizing the tonsillobiventral fissure for accessing the supratonsillar recess. The supratonsillar approach is a valuable alternative to transcerebellar or telovelar approaches that minimizes tissue transgression and brain retraction.

**Figure 1:**
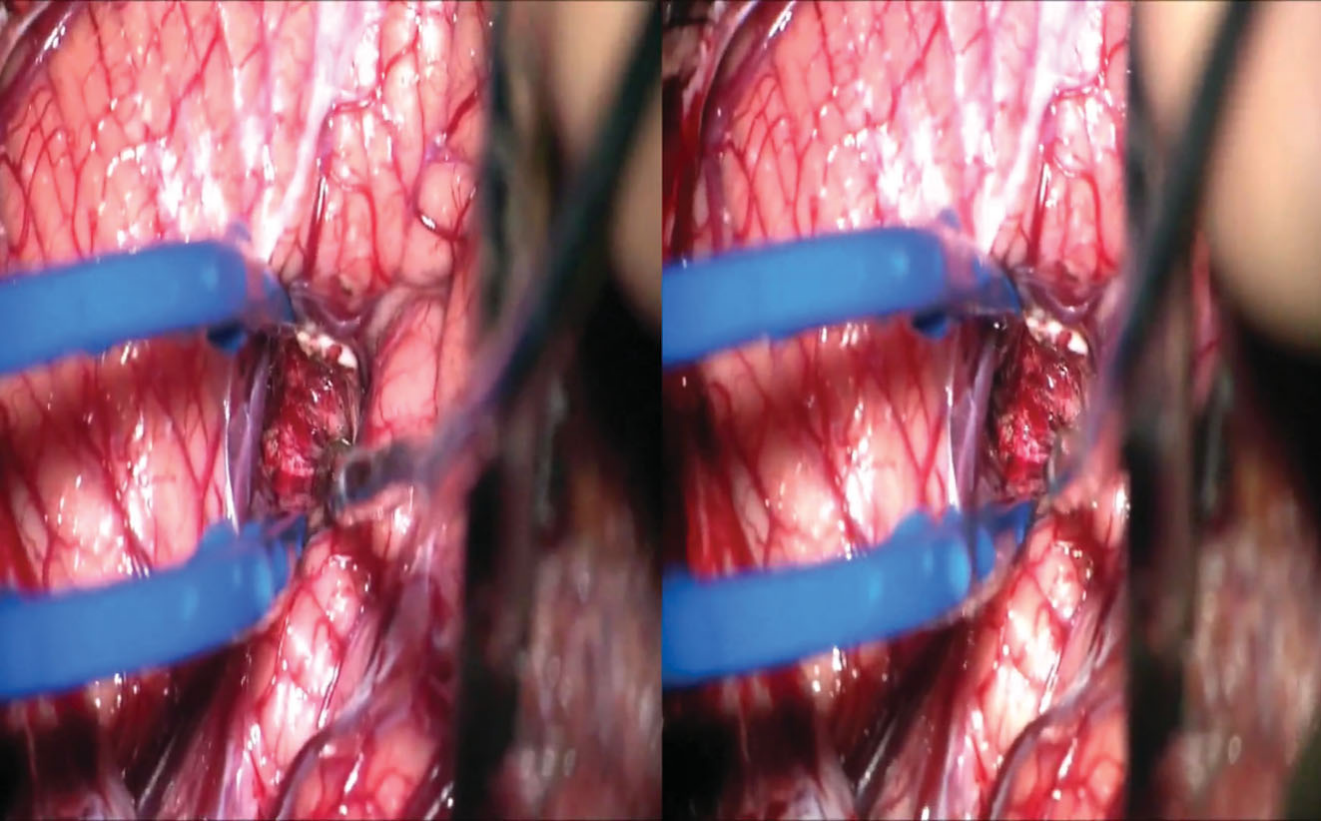
Watch now at https://academic.oup.com/ons/article-lookup/doi/10.1093/ons/opx030

## Disclosure

The authors have no personal, financial, or institutional interest in any of the drugs, materials, or devices described in this video.
